# Rare pancreatic metastasis of undifferentiated pleomorphic sarcoma originating from the pelvis: A case report

**DOI:** 10.1016/j.ijscr.2020.02.041

**Published:** 2020-02-21

**Authors:** Manato Ohsawa, Yoshihiro Mikuriya, Koji Ohta, Minoru Tanada, Noriaki Yamamoto, Norihiro Teramoto, Masahiro Kiyono, Shinsuke Sugihara

**Affiliations:** a Department of Surgery, National Hospital Organization Shikoku Cancer Center, 160 Minamiumemotomachikou, Matsuyama-shi, Ehime, Japan; b Department of Pathology, National Hospital Organization Shikoku Cancer Center, 160 Minamiumemotomachikou, Matsuyama-shi, Ehime, Japan; c Department of Orthopedic Surgery, National Hospital Organization Shikoku Cancer Center, 160 Minamiumemotomachikou, Matsuyama-shi, Ehime, Japan

**Keywords:** CT, computed tomography, FDG, fluorodeoxyglucose, MRI, magnetic resonance imaging, PET, positron emission tomography, UPS, undifferentiated pleomorphic sarcoma, Undifferentiated pleomorphic sarcoma, Pancreas, Metastasis

## Abstract

**Introduction:**

Undifferentiated pleomorphic sarcoma (UPS) is a reclassification of malignant fibrous histiocytoma by the World Health Organization in 2002. UPS, the most common soft tissue sarcoma reported in adults, mostly recurs as lung disease. Pancreatic metastasis of UPS is extremely rare. We present a rare case of pelvic UPS with pancreatic metastasis.

**Presentation of case:**

A 69-year-old man was identified as having mediastinal lymphadenopathy on follow-up computed tomography (CT), 2 years after undergoing surgery for gastric adenocarcinoma (pT4aN3M0/IIIC). Subsequent positron emission tomography–CT (PET/CT) indicated pelvic lesions and magnetic resonance imaging (MRI) showed multiple tumors of the left pubis and femur. Histopathology of diagnostic thoracoscopic lymph node dissection and CT-guided needle biopsy of the left pubic lesion showed UPS. Systemic chemotherapy and targeted molecular therapy reduced multiple pelvic and metastatic tumors. Left pubic primary lesion contraction was achieved with intensity-modulated radiation therapy. CT performed 4 years after treatment initiation showed a 40-mm pancreatic head mass. Lesions other than the pancreatic tumor were in remission, and a pancreatoduodenectomy was performed. Histological analysis confirmed pancreatic metastasis of anaplastic pleomorphic sarcoma.

**Discussion:**

Reports of pelvic UPS with pancreatic metastasis, as that of the present case, are extremely rare. UPS is malignant potential tumor, and complete excision is the first treatment option, while the usefulness of chemotherapy or radiation therapy remains uncertain.

**Conclusions:**

Complete resection is vital for local control in pancreatic metastasis of UPS. Sites of recurrence are rare; hence, patients must be carefully followed up.

## Highlights

• Pancreatic metastasis of UPS is extremely rare.
• We report the clinical management of pancreatic metastasis of pelvic UPS.
• Complete resection of pancreatic metastasis of UPS is vital for local control.

## 1 Introduction

This case is reported in line with the SCARE criteria [1]. Undifferentiated pleomorphic sarcoma (UPS) is a reclassification of malignant fibrous histiocytoma by the World Health Organization in 2002 [2]. UPS, the most common soft tissue sarcoma in adults, commonly occurs in soft tissues and long bones of the extremities and trunk. UPS is a malignant potential tumor, and most patients with recurrence present with lung disease [3]. Pancreatic metastasis of UPS is extremely rare [4]. Herein, we present a rare case of pelvic UPS with metastasis to the pancreas.

## 2 Presentation of case

**Fig. 1:**
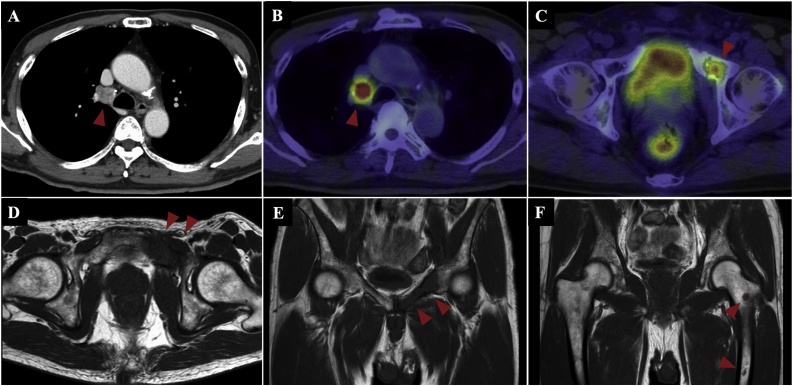
Imaging of primary and metastatic lesions. Arrowheads indicate the tumor. (A) Contrast-enhanced computed tomography reveals mediastinal lymphadenopathy. (B) This lymph node demonstrates high fluorodeoxyglucose uptake on positron emission tomography. (C) A high level of fluorodeoxyglucose accumulation is observed in the pubic bone. (D) T1-weighted magnetic resonance imaging shows multiple tumors of the pubic bone and femur, (E) coronal section, and (F) sagittal section.

A 69-year-old man was identified as having mediastinal lymphadenopathy on follow-up computed tomography (CT), 2 years after surgery for gastric adenocarcinoma (pT4aN3M0/IIIC) at our hospital (Fig. 1A). The mediastinal lymph nodes demonstrated high fluorodeoxyglucose (FDG) uptake on positron emission tomography (PET) (Fig. 1B). Furthermore, a high accumulation of FDG was observed in the pelvis (Fig. 1C). Magnetic resonance imaging (MRI) showed multiple tumors of the left pubis and femur (Fig. 1D–F). We suspected lymph node metastasis of gastric cancer. However, a diagnostic thoracoscopic lymphadenectomy and subsequent pathology confirmed UPS. A CT-guided needle biopsy of the left pubic lesion revealed similar pathological results. The diagnosis was UPS with a primary lesion involving the left pubis, and multiple metastases to the pelvic bones, femur, and mediastinal lymph nodes.

After consultation with an orthopedic surgeon, treatment was initiated. The patient received doxorubicin and cisplatin as systemic chemotherapy and denosumab as molecular targeted therapy for receptor activator of nuclear factor κB ligand. The doxorubicin/cisplatin regimen included intravenous (IV) infusion of cisplatin at a dose of 80 mg/m^2^ on day 1 and IV infusion of doxorubicin at a dose of 20 mg/m^2^ on days 1–3. Eight such courses were performed at 3-week intervals. Denosumab (dose, 120 mg) was injected subcutaneously every 4 weeks. After therapy, MRI showed shrinkage of multiple metastatic tumors. In addition, intensity-modulated radiotherapy (72.6 Gy in 30 fractions) was delivered to the primary lesion in the left pubic bone. Primary tumor shrinkage was achieved and tumor remission was obtained. Denosumab was administered every 4 weeks to maintain the tumor shrinkage.

**Fig. 2:**
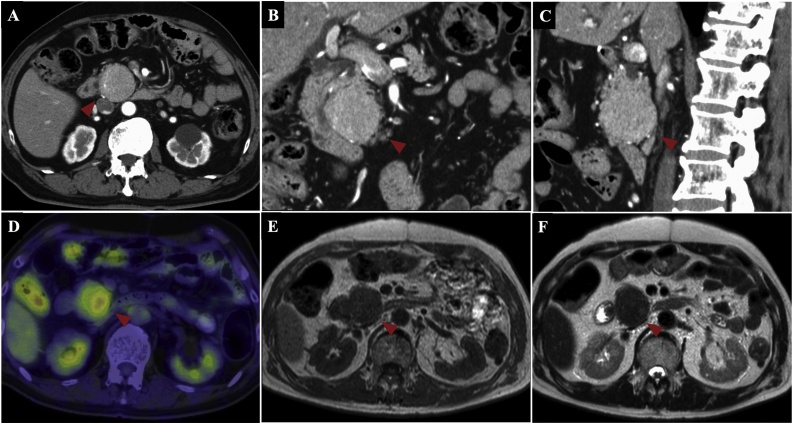
Contrast-enhanced computed tomography reveals a 40-mm well-defined solid mass in the pancreatic head (A, B: Coronal section, C: Sagittal section). Arrowheads indicate the tumor. The solid mass demonstrates high fluorodeoxyglucose uptake on positron emission tomography (D). On both T1- and T2-weighted magnetic resonance imaging, the mass is marginated with a low intensity. There is no apparent invasion of other organs (E, F).

**Fig. 3:**
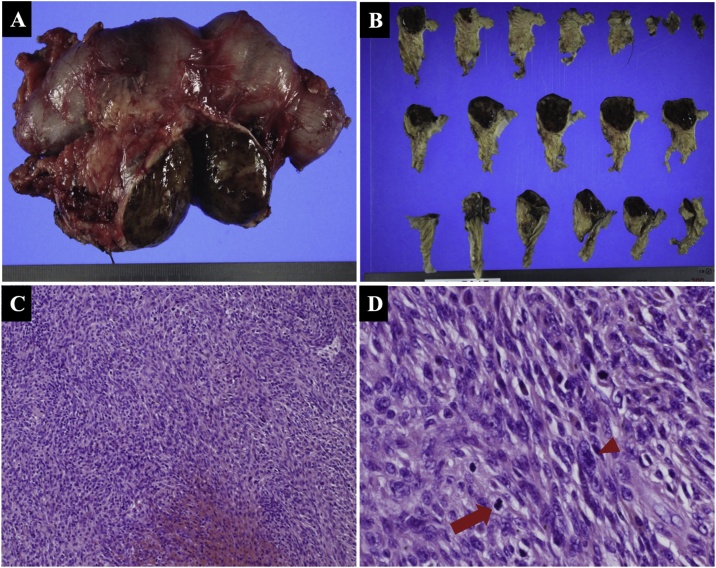
Macroscopic examination of the resected specimen reveals a 50-mm tumor in the pancreatic head, and the cut surface of the tumor is solid dark brown (A, B). Microscopic examination demonstrates that the tumor is confined to the pancreatic tissue with histiocytic atypical cell proliferation in bundles and a storiform pattern, and some large atypical nuclei (arrowhead) and mitotic images (arrow) are found (hematoxylin-eosin staining, C: ×100, D: ×400).

**Fig. 4:**
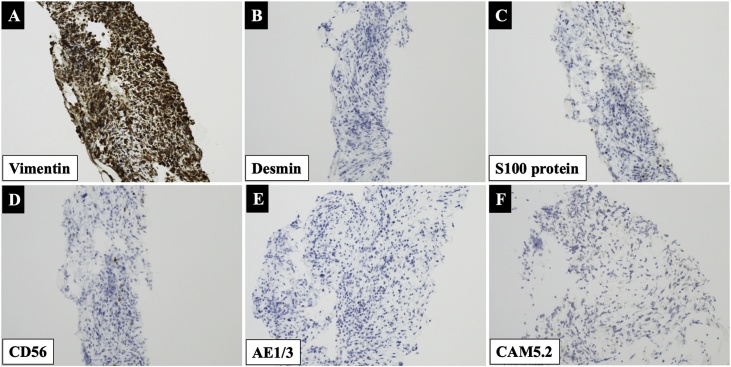
Immunostaining shows (A) vimentin-positive, (B) desmin-negative, (C) S100 protein-negative, (D) CD 56-negative, (E) AE1/AE3-negative, and (F) CAM5.2-negative cells.

Two years after tumor remission for UPS was obtained, follow-up CT showed a 40-mm mass in the pancreatic head. Pancreatic metastasis of UPS was suspected. Notably, the patient had no pertinent family history. A surgeon was consulted regarding the pancreatic mass. The patient had no chief complaints. On physical examination, the abdomen was soft, not distended, and non-tender. Biochemistry test results revealed slight elevation in the level of serum carcinoembryonic antigen (5.8 ng/mL) and a normal level of carbohydrate antigen 19-9. Upper gastrointestinal endoscopy and biopsy were not feasible because of previous total gastrectomy and Roux-en-Y reconstruction. Contrast-enhanced CT revealed a 40-mm, well-defined, solid mass in the pancreatic head (Fig. 2A–C). There were no other obvious metastatic lesions. The solid mass demonstrated high FDG uptake on PET (Fig. 2D). Both T1- and T2-weighted MRI showed one low-intensity, marginated mass. There was no apparent invasion of other organs (Fig. 2E, F). Based on the above findings, the patient was diagnosed with pancreatic metastasis of UPS. Since the primary tumor and multiple pelvic metastases were controlled after treatment, surgery for the pancreatic head tumors was planned. Pancreaticoduodenectomy was performed, and complete tumor resection was achieved. On the basis of the previous total gastrectomy and Roux-en-Y reconstruction, the afferent loop including the anastomosis was excised, the jejunum of the efferent loop was raised, and pancreaticojejunostomy and bile duct jejunostomy were performed. The reconstruction was completed by anastomosing the jejunum on the esophagus side and the jejunum raised from the original Y-loop anastomosis. The operative time was approximately 418 min. Intraoperative blood loss was approximately 1160 mL. Macroscopic examination of the resected specimen revealed a 50-mm tumor in the pancreatic head (Fig. 3A). The cut surface of the tumor was solid dark brown (Fig. 3B). Microscopic examination demonstrated that the tumor was confined to the pancreatic tissue with histiocytic atypical cells that proliferated in bundles and a storiform pattern. Some large atypical nuclei and mitotic images were also found (Fig. 3C, D). Immunostaining showed vimentin (+), desmin (−), S100 protein (−), CD56 (−), cytokeratin AE1/AE3 (−), anti-cytokeratin CAM5.2 (−) (Fig. 4A–F). These results were similar to the pathological results of the pubic needle biopsy. Consequently, the diagnosis of pancreatic metastasis of UPS was confirmed. No malignant cells were detected in the resection stump. The patient recovered uneventfully and was discharged 30 days after surgery. He is currently undergoing outpatient follow-up 2 months after surgery.

## 3 Discussion

UPS, the most common adult soft tissue sarcoma, commonly occurs in soft tissues and long bones of the extremities and trunk with an incidence of 1 case per 100,000 individuals [3]. It commonly affects individuals older than 40 years of age, is slightly more common among men, and starts as a primary tumor in 70% of cases or arises from preexisting conditions such as radiation therapy of the affected area in 30% of cases [5,6]. A contrast-enhanced CT performs non-uniform imaging from equal absorption to low absorption. On MRI, the lesions show high signal intensity on T2-weighted images and low to equal signal intensity on T1-weighted images [7]. In our patient, the pancreatic metastatic lesion showed low absorption areas on both T1- and T2-weighted images. Complete excision of the tumor is the first choice of treatment. No definitive opinion exists of the usefulness of chemotherapy or radiation therapy [8]. However, adjuvant chemotherapy is considered useful [5,6,9]. In our patient, lesions other than the pancreatic head tumor were controlled by chemotherapy, molecular targeted therapy, and radiotherapy. Surgical resection, which has the highest therapeutic effect, was selected for local control of the pancreatic head tumor. No adjuvant chemotherapy was used because the limit dose had already been administered in doxorubicin/cisplatin therapy.

The typical pathology of UPS shows undifferentiated histiocytic atypical cells proliferating in a disorderly manner with atypical spindle-shaped cells without a specific arrangement tendency. Although tumor cells may be arranged in a storiform pattern, as seen in our case, they are not specific to UPS. On immunostaining, UPS shows characteristic positive staining for histiocytic markers (vimentin, CD68, and α1-antichymotrypsin), a reason why such tumors were identified as malignant fibrous histiocytoma in the past. Results of staining for TTF-1, S-100 protein, desmin, actin, myoglobin, caldesmon, D2-40, and calretinin are usually negative. Our patient was negative for keratin staining (AE1/AE3 and CAM5.2). However, in some cases, keratin staining may be positive, which makes it challenging to differentiate sarcomatoid carcinomas from UPS. Stronger cytokeratin immunoreactivity with more differentiated carcinomatous elements and immunoreactivity for epithelial markers (such as P63) can be helpful in the diagnosis of sarcomatoid carcinomas [[10], [11], [12]].

UPS is a malignant potential tumor, with a 2-year survival rate of 60% and 5-year survival rate of 14–36% [3,13]. The local recurrence rate after resection ranges from 13% to 52%, and the distant metastasis rate ranges from 31% to 35% of patients despite aggressive surgery [3,14,15]. Most recurrences are said to occur within 2 years after treatment [16]. Large tumor size, deep invasion, and microscopically positive surgical margins are considered to be factors that affect local recurrence [5]. Most patients with recurrence present with lung disease (82%). Metastasis to extrapulmonary locations is not frequent and can occur in lymph nodes (32%) and in the bones and liver (15%) [3]. Reports of cases of pancreatic metastasis of UPS are scarce. A PubMed database search of English articles yielded only one result [4]. Even before 2002, when reclassified from malignant fibrous histiocytoma to UPS, there were only three reported cases of pancreatic metastasis; one of the patients underwent distal pancreatectomy [[17], [18], [19]].

Large tumor size, tumor presentation (recurrent), and invasion to important structures (bone, vessel, or nerve) are considered crucial prognostic factors [20]. Pancreaticoduodenectomy was considered the most suitable treatment option for the patient in our case.

## 4 Conclusion

Pancreatic metastasis of UPS is extremely rare and requires further research. Complete resection is of utmost importance for local control of this malignant potential tumor. Sites of recurrence are rare; hence, patients should undergo careful follow-up.

## Funding

The authors declare that this study was not funded externally.

## Ethical approval

As a case report without Protected Health Information, no ethics approval was required for this project.

## Consent

Written informed consent was obtained from the patient for the publication of this case report and any accompanying images. A copy of the written consent is available for review by the Editor-in-Chief of this journal.

## Author contribution

MO, YM, and KO drafted the article and performed the literature search. MO, YM, KO, MT, NY, NT, MK, and SS contributed to patient care and participated in the critical revision of the article. All authors have read and approved the final article.

## Registration of research studies

This is a case report.

## Guarantor

Manato Ohsawa.

## Provenance and peer review

Not commissioned, externally peer-reviewed.

## Declaration of Competing Interest

The authors declare no conflicts of interest.
